# Comparison of Rapid Cognitive Screen against Montreal Cognitive Assessment in screening for cognitive impairment in the old and old‐old

**DOI:** 10.1111/psyg.12841

**Published:** 2022-05-16

**Authors:** Matthew Zhixuan Chen, Yiong Huak Chan, Michael Wai Kit Wong, Reshma Aziz Merchant

**Affiliations:** ^1^ Division of Geriatric Medicine, Department of Medicine National University Hospital Singapore Singapore; ^2^ Biostatistics Unit, Yong Loo Lin School of Medicine National University of Singapore Singapore Singapore; ^3^ Department of Medicine, Yong Loo Lin School of Medicine National University of Singapore Singapore Singapore

**Keywords:** cognitive impairment, Montreal Cognitive Assessment, Rapid Cognitive Screen, validation

## Abstract

**Background:**

The Montreal Cognitive Assessment (MoCA) was developed as a screening tool for mild cognitive impairment (MCI). Given the need for a rapid screening test in settings such as primary care, we compare the validity of the Rapid Cognitive Screen (RCS) against the MoCA, and determine cut‐off scores in the old and old‐old.

**Methods:**

Cross‐sectional study involving community‐dwelling ‘old’ (65 to 79 years old) and ‘old‐old’ (≥ 80 years old) without dementia. Cognitive impairment was defined by MoCA score 17 to 22. Validation was done using the receiver operating characteristic (ROC) curve analysis: area under the curve (AUC), sensitivity (Sn), and specificity (Sp).

**Results:**

Of the 183 participants (mean age 72.1 ± 5.2 years),15.8% (*n* = 29) were classified as cognitively impaired. The overall ROC curve had an AUC of 0.82 (95% CI 0.75–0.90, *P* < 0.01) with an optimal cut‐off of 7/8 on RCS (Sn 0.77, Sp 0.72). The ‘old’ and ‘old‐old’ group had AUC of 0.82 (95% CI 0.74–0.91, *P* < 0.01) with 8/9 as optimal cut‐off (Sn 0.51, Sp 0.96) and AUC of 0.85 (95% CI 0.66–1.03, *P* < 0.01) with 7/8 as optimal cut‐off (Sn 0.71, Sp 1.00) respectively. In multivariate analysis, age was associated with 0.05 (95% CI ‐0.10‐0.00, *P* < 0.04) point decrement, while >6 years of education was associated with 0.82 (95% CI 0.32–1.33, *P* < 0.01) point increment in RCS scores.

**Conclusion:**

The three‐item RCS is quick and easy to administer. Although RCS met the criterion for good validity against MoCA in predicting cognitive impairment, its utility as a first‐line screening tool needs to be further validated in a large‐scale population study.

## INTRODUCTION

The prevalence of dementia has more than doubled between 1990 and 2016, and is projected to increase from 43.8 million in 2016 to 82 million in 2030 mainly due to unprecedented rise in the number of older adults and increasing lifespan.^1^, ^2^ In 2015, the cost of caring for persons with dementia was estimated to be US$818 billion, and is estimated to increase to US$2 trillion by 2030.^3^ As there is no cure for dementia, and dementia onset may be delayed through multi‐domain interventions focusing on physical inactivity, smoking, obesity, hearing impairment, education, diabetes, hypertension and education, early screening for mild cognitive impairment (MCI) is important in older adults at risk.^4^, ^5^, ^6^ MCI is a clinical and neuropsychological syndrome where individuals demonstrate cognitive impairment beyond normal ageing but with intact or minimal impairment of functional abilities not amounting to dementia,^5^, ^7^ with an annual conversion rate to Alzheimer's dementia observed in 10.2% to 33.6%.^8^ However, reversion to normal cognition has also been observed in 10% to 40% over 5 years in various studies.^9^, ^10^, ^11^, ^12^ Prevalence of MCI in the general population is difficult to estimate due to varying diagnostic criteria leading to a wide range of estimates between 3% to 42%, and strongly associated with increased age and lower educational level.^5^, ^13^

With increasing attention to age‐friendly health systems, and recommendations on upstream screening to improve health and well‐being of older adults, fast and practical tools such as the Rapid Geriatric Assessment (RGA), which can be administered by any trained person, have been developed.^14^, ^15^, ^16^ The Rapid Cognitive Screen (RCS) which is part of the RGA takes less than 3 min to administer and has been validated in different countries.^17^, ^18^ Widely used screening tests for cognitive impairment such as the Mini‐Mental State Examination (MMSE) have inherent limitations such as ceiling effects and the absence of executive testing.^19^, ^20^ The Montreal Cognitive Assessment (MoCA) is regarded to be superior to the MMSE in detecting cognitive impairment for those at higher risk of incident dementia as it can assess executive function, higher‐level language, and complex visuospatial processing with less ceiling effect,^21^, ^22^ but both tools take longer times to administer which make them less desirable for use in certain settings such as primary care or in the community. Briefer screening tests such as the Mini‐cog and clock drawing test (CDT) are available as well, but may not have good sensitivity (Sn) and specificity (Sp) to identify MCI.^23^

The RCS includes three items from the Veterans Affairs Saint Louis University Mental Status examination. It includes recall of five words (testing recall), a CDT (testing visuospatial function), and the ability to remember a story and convert the fact that Kuala Lumpur is in Malaysia, or Chicago is in Illinois (testing insight and executive function).^24^ It is easy to administer and takes less than 3 min, with a cut‐off of 7/8 suggestive of cognitive dysfunction. Its Sn = 0.87 and Sp = 0.70 are superior to that of CDT plus recall (Sn = 0.62, Sp = 0.62).^17^

Through our study, we aim to compare the validity of the RCS against the MoCA in those with possible cognitive impairment, and determine whether cut‐off scores in the ‘old’ and ‘old‐old’ should be adjusted for improved performance.

## METHODS

Community‐dwelling older adults aged ≥65 years were screened and recruited for a frailty prevention intervention study from two primary care centres in the western region of Singapore between 2019 to 2021. Their primary care physician or nurse signed a slip to certify that they did not have dementia and were able to understand and sign the consent form. Cognitive impairment was defined as MoCA scores between 17 and 22.^25^, ^26^ Those who screened positive on the Geriatric Depression Scale (score >5) were excluded from analysis.^27^

Participants who were at least pre‐frail but ambulant were invited for an interview, and data were collected on socio‐demographics, education background, functional status, sarcopenia, chronic diseases, medications, depression and cognition. The sessions were conducted by trained research staff and relied mainly on self‐report. Cognition was assessed using RCS and MoCA. RCS is comprised of three sections including five‐item recall (5 points), clock drawing (4 points) and a story recall (1 point). The maximum is 10 points. The MoCA assesses multiple domains including attention, concentration, executive functions, memory, language, visuospatial skills, abstraction, calculation and orientation, and a cut‐off score of 17–22 was used to define cognitive impairment.^22^ Barthel Index and Lawton's Instrumental Activities of Daily Living scale were used for assessing functional status.^28^, ^29^ Sarcopenia was screened using the SARC‐F tool (Strength, Assistance in walking, Rising from a chair, Climbing stairs, and Falls). Maximal score is 10 and ≥4 is indicative of sarcopenia.^30^ Polypharmacy referred to the use of five or more prescribed medications daily.

All research procedures and purposes of the study were explained in detail and written consent was obtained from all recruited participants. The study complies with the Declaration of Helsinki ethical principles and its protocol was approved by the National Healthcare Group, Domain Specific Review Board, Singapore.

### Statistical analysis

The data were analysed using IBM SPSS Version 26.0. Categorical variables are presented as frequencies (%) while continuous variables were stated as means ± SD. Chi‐squared test and independent *t*‐test were used for the categorical and continuous variables respectively to determine significant differences between cognitively impaired and non‐cognitively impaired groups.

Receiver operating characteristic (ROC) curve analysis was used to evaluate the performance of RCS in discriminating participants with cognitive impairment from those without cognitive impairment. Area under the curve (AUC) scores were reported for the whole sample and two subgroups: by participants' age (at least 80 years and above, or below) and by education (more than 6 years of education, or below). The optimal RCS cut‐off scores were determined using Youden's Index. Sn, Sp, as well as positive predictive value (PPV) and negative predictive value (NPV) of the optimal cut‐off scores were also calculated.

Relative contributions of gender, age, and education on variations in RCS score were estimated using general linear modelling. The mean differences between groups were reflected as B‐coefficients with 95% confidence intervals. Statistical significance was determined as *P* < 0.05.

## RESULTS

Our study consisted of 183 community‐dwelling adults. Mean age was 72.1 ± 5.2 years. Mean educational level was 8.0 ± 4.0 years. Women made up 51.9% of the cohort and 83.6% were of Chinese ethnicity. About one in seven (15.8%) of the participants had cognitive impairment (Table 1). Among women, 16.8% had cognitive impairment compared to 14.8% in men. Among the Chinese ethnic group, 12.4% had cognitive impairment compared to 28.6% Indian and 43.8% Malay ethnic groups. Participants with cognitive impairment were generally older (73.4 ± 5.6 years) and had lower educational levels (5.2 ± 4.1 educational years) than those without cognitive impairment (71.8 ± 5.1 years; 8.5 ± 3.8 educational years). Those with cognitive impairment had significantly higher prevalence of sarcopenia and slower gait speed than those without, 28.6% versus 10.5% (*P* = 0.02) and 0.78 ± 0.25 versus 0.92 ± 0.23 m/sec (*P* < 0.01) respectively. Mean MoCA and RCS scores were significantly lower in the group with cognitive impairment than the non‐cognitively impaired group, 20.4 ± 1.3 versus 26.9 ± 2.0 and 6.2 ± 1.8 versus 8.3 ± 1.5 respectively.

**Table 1:** Characteristics of participants

Variable	All *n*=183 (100)	No CI [MoCA 23‐30] *n*=154 (84.2)	CI [MoCA 17‐22] *n*=29 (15.8)	*p* value
Gender^a^				0.70
Male, *n*	88 (48.1)	75 (85.2)	13 (14.8)	
Female, *n*	95 (51.9)	79 (83.2)	16 (16.8)	
Ethnicity^a^				**0.02**
Chinese, *n*	153 (83.6)	134 (87.6)	19 (12.4)	
Malay, *n*	16 (8.7)	9 (56.2)	7 (43.8)	
Indian, *n*	14 (7.7)	10 (71.4)	4 (28.6)	
Age, *years*	72.1±5.2	71.8±5.1	73.4±5.6	0.13
Education, *years*	8.0±4.0	8.5±3.8	5.2±4.1	**<0.01**
Living alone, *n*	13 (7.1)	11 (7.1)	2 (6.9)	0.64
Number of ADL impairments (≥1), *score*	1.3±1.0	1.2±0.8	1.8±2.0	0.47
Number of IADL impairments (≥1), *score*	2.0±1.7	1.9±1.5	3.3±3.3	0.49
Gait speed, *metre/ second*	0.90±0.24	0.92±0.23	0.78±0.25	**<0.01**
Subjective memory complains, *n*	33 (18.2)	26 (17.0)	7 (25.0)	0.31
Sarcopenia, *n*	19 (13.1)	13 (10.5)	6 (28.6)	**0.02**
Hypertension, *n*	132 (73.3)	111 (73.5)	21 (72.4)	0.90
Diabetes, *n*	98 (54.1)	82 (53.9)	16 (55.2)	0.90
Hyperlipidaemia, *n*	154 (85.1)	131 (86.2)	23 (79.3)	0.34
Polypharmacy, *n*	58 (32.0)	50 (32.9)	8 (27.6)	0.58
MoCA, *score*	25.8±3.0	26.9±2.0	20.4±1.3	**<0.01**
RCS, *score*	8.0±1.7	8.3±1.5	6.2±1.8	**<0.01**

Bold indicates *p* < 0.05; Values are n (%), otherwise Mean ± Standard Deviation (SD).

^a^ Row %, otherwise column %.

Abbreviations: CI, cognitive impairment; ADL, activities of daily living; IADL, instrumental activities of daily living; EQ‐VAS, euroqol visual analogue scale; MoCA, montreal cognitive assessment; RCS, rapid cognitive screen.

The prevalence of cognitive impairment was higher among those with 6 or less years of education than those with more than 6 years of education (23.0% vs. 11.1%), as well as those in the ‘old‐old’ group (defined as age ≥ 80 years) than those in the ‘old’ group (defined as age 65 to 79 years) (17.6% vs. 15.7%). Mean RCS scores between participants with and without cognitive impairment were significantly different (*P* < 0.01) across all subgroups except those in the ‘old‐old’ group (*P* = 0.07).

**Figure 1:**
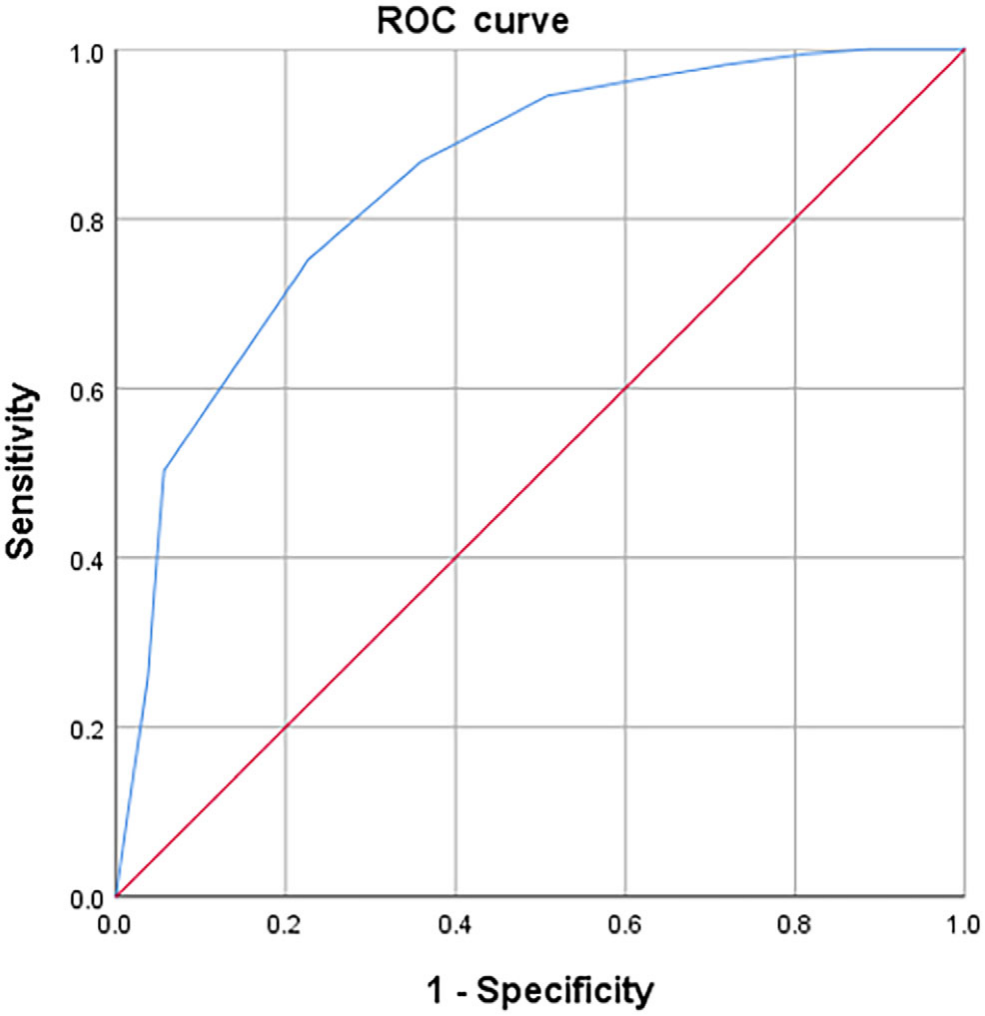
Rapid Cognitive Screen receiver operating characteristic (ROC) curve for mild cognitive impairment on the Montreal Cognitive Assessment, whole population.

**Figure 2:**
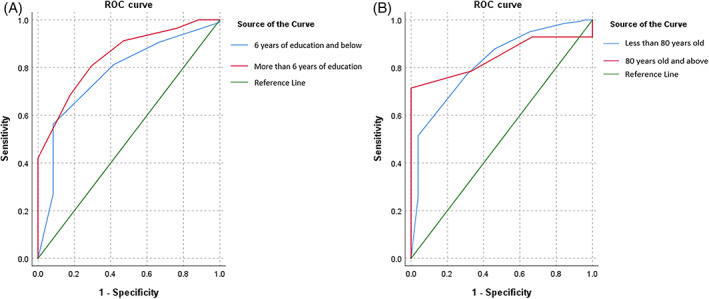
(A) Rapid Cognitive Screen (RCS) receiver operating characteristic (ROC) curves for mild cognitive impairment (MCI) on the Montreal Cognitive Assessment (MoCA), by education. (B) RCS ROC curves for MCI on MoCA, by age.

Comparing the validity of RCS against MoCA for the entire study sample, the ROC curve had an AUC of 0.82 (95% CI 0.75–0.90, *P* < 0.01) with an optimal cut‐off of 7/8 on RCS (Sn 0.77, PPV 0.37, Sp 0.72, NPV 0.94) (Fig. 1). The ROC curves of those with ≤6 years of education and >6 years of education had AUC of 0.85 (95% CI 0.75–0.94, *P* < 0.01) and 0.77 (95% CI 0.63–0.91, *P* < 0.01) respectively (Fig. 2) (Table 2). Their respective optimal cut‐offs on RCS were 6/7 (Sn 0.81, PPV 0.52, Sp 0.71, NPV 0.90) and 8/9 (Sn 0.56, PPV 0.21, Sp 0.92, NPV 0.98). The ROC curve for participants in the ‘old’ group had AUC of 0.82 (95% CI 0.74–0.91, *P* < 0.01) on its ROC curve with 8/9 as its optimal cut‐off (Sn 0.51, PPV 0.27, Sp 0.96, NPV 0.99) (Fig. 2b) (Table 2). Those in the ‘old‐old’ group had AUC of 0.85 (95% CI 0.66–1.03, *P* < 0.01) with 7/8 as its optimal RCS cut‐off (Sn 0.71, PPV 0.43, Sp 1.00, NPV 1.00).

**Table 2:** Test performance of RCS for CI (MoCA 17‐22)

	All			6 years of education and below			More than 6 years of education			‘Old’ (Age 65 to 79 years)			‘Old‐old’ (Age 80 years and above)		
	No CI	CI	*p*/Youden's Index	No CI	CI	*p*/Youden's Index	No CI	CI	*p*/Youden's Index	No CI	CI	*p*/Youden's Index	No CI	CI	*p*/Youden's Index
Participants (row %)	154 (84.2)	29 (15.8)		57 (77.0)	17 (23.0)		96 (88.9)	12 (11.1)		140 (84.3)	26 (15.7)		14 (82.4)	3 (17.6)	
Mean RCS Score ± SD	8.3±1.5	6.2±1.8	**<0.01**	8.0±1.7	5.5±1.7	**<0.01**	8.5±1.4	7.2±1.3	**<0.01**	8.4±1.5	6.2±1.8	**<0.01**	8.1±1.8	6.0±1.0	0.07
AUC (95% CI)	0.82 (0.75‐0.90)		**<0.01** ^**a**^	0.85 (0.75‐0.94)		**<0.01** ^**a**^	0.77 (0.63‐0.91)		**<0.01** ^**a**^	0.82 (0.74‐0.91)		**<0.01** ^**a**^	0.85 (0.66‐1.03)		**<0.01** ^**a**^
Optimal Cut‐off	7/8		0.49	6/7		0.51	8/9		0.48	8/9		0.48	7/8		0.71
Sensitivity/PPV	0.77/0.37			0.81/0.52			0.56/0.21			0.51/0.27			0.71/0.43		
Specificity/NPV	0.72/0.94			0.71/0.90			0.92/0.98			0.96/0.99			1.00/1.00		

Bold indicates significance.

^a^ Null hypothesis: true area = 0.5.

Abbreviation: RCS, Rapid Cognitive Screen; CI, cognitive impairment; *p*, *p* value; SD, standard deviation; AUC, area under curve; 95% CI, 95% confidence interval.

The relative contributions of gender, age, and education years to variances in RCS scores were examined in Table 3. Age and education were predictors of RCS scores, but with varying magnitudes and directions. For every yearly increase in age, participants were associated with 0.05 (95% CI –0.10 to 0.00, *P* < 0.04) point decrement, while those with more than 6 years of education were associated with 0.82 (95% CI 0.32–1.33, *P* < 0.01) point increment in RCS scores. Gender was not an independent predictor for RCS.

**Table 3:** Regression model estimates of the relative contributions to RCS score

	B‐coefficient (95% CI)	*P*	*t*	η^2^
Female gender, (ref. male)	0.07 (−0.42‐0.56)	0.78	0.28	<0.01
Age (in years)	−0.05 (−0.10‐0.00)	**0.04**	−2.1	0.02
More than 6 years of education, ref.^6^ years of education and below	0.82 (0.32‐1.33)	**<0.01**	3.23	0.06

Bold indicates significance.

Abbreviation: RCS, Rapid Cognitive Screen; *p*, *p* value; 95% CI, 95% confidence interval; *t*, *t* value.

η^2^, partial eta squared (effect size).

## DISCUSSION

Our study is the first to demonstrate the performance of the RCS against MoCA for cognitive impairment in screening for cognitive impairment among the ‘old’ and ‘old‐old’ multi‐ethnic populations. While the construct validity was described in the original literature, we were able to show that the RCS had external validity and found it to have acceptable specificity and sensitivity expected of a screening tool when compared with MoCA. While MoCA has been found to be superior in screening for cognitive impairment, the optimal cut‐off has been a constant debate, especially in a multi‐ethnic community with diverse educational levels.^31^ MoCA cut‐off score of 23 rather than the initial recommended score of 26 lowers the false positive rates and has shown to improve diagnostic accuracy.^32^, ^33^ The benefits of the RCS over the MoCA lies in the ease of administration and shorter time of administration making it a practical tool for first‐line screening at the community level.

**Figure 3:**
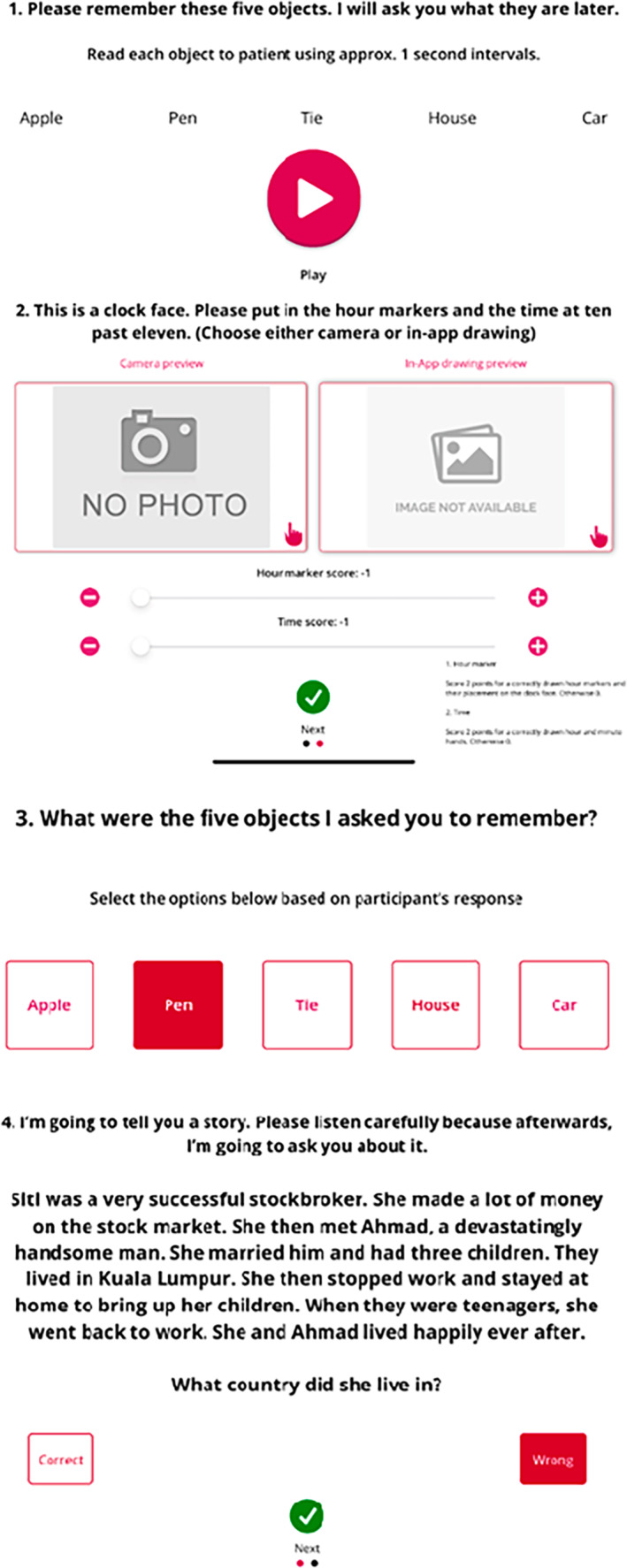
Rapid Cognitive Screen iPad and iPhone application.

The RCS is embedded in the RGA which together with the FRAIL scale, SARC‐F, and Short Nutritional Assessment Questionnaire can serve as a useful tool for busy primary care physicians to identify older adults at risk of frailty, sarcopenia, anorexia of ageing and cognitive impairment.^16^, ^34^ Accessibility of this valuable set of tools has been enhanced further in the form of a mobile application (Fig. 3), which may also allow individuals to perform a self‐assessment and receive personalised advice on cognitive, physical, and dietary interventions.^35^

While our study was not truly representative of community‐dwelling older adults, the prevalence of cognitive impairment was 15.8%, which falls within the 3% to 42% prevalence worldwide, and was significantly associated with increasing age and lower educational level.^5^, ^13^, ^36^ Interestingly, we found that the RCS performed well when compared against MoCA in the ‘old‐old’, which has not previously been described. RCS includes three domains of recall, visuospatial function and executive function/insight, whereas MoCA includes an extensive range of domains as described earlier. CDT and recall have independently shown to be good predictors of cognitive decline in stroke patients and the general population.^37^, ^38^ In a systematic review, the MMSE had sensitivity and specificity of 0.71 and 0.74 respectively in detecting MCI. The sensitivity and specificity of MoCA was 0.83 and 0.75 respectively, and recall tests showed the best diagnostic performance with sensitivity and specificity of 0.89 and 0.84 respectively.^38^

RCS is in no way diagnostic for cognitive impairment but can be used as a first‐line screening tool at the population level and further supplemented with neuroimaging and biomarkers to increase the predictive power for those who will ultimately progress to dementia.^39^ Early identification of cases at risk of progression can provide the golden opportunity for active intervention including lifestyle changes, participation in risk reduction strategies which can delay the cognitive, functional and behavioural decline, and advanced care planning.

While studies recommend subjective cognitive complaints to guide early case finding approaches, there were no significant differences in our population with cognitive impairment. The possible explanations may include recall bias or under‐reporting by certain ethnic groups. Our prior published study did highlight that the Chinese ethnic group over‐reports, and Malay ethnic group under‐reports subjective cognitive decline.^40^ The prevalence of cognitive impairment was lower in the Chinese ethnic group compared with the Malay ethnic group.

The prevalence of sarcopenia was significantly higher in those with cognitive impairment which is consistent with a growing body of evidence in recent years.^41^, ^42^, ^43^ Brain‐derived neurotrophic factor released by contracting muscle regulates neurogenesis and synaptic plasticity with beneficial effect on overall cognition. The underlying mechanism or causal correlation is an ongoing area of research with one of the most recent studies suggesting that poor muscle function, rather than reduced lean muscle mass, may be responsible for late‐life cognitive impairment.^44^, ^45^ Both handgrip strength and slow gait speed are associated with poor cognitive function, which are also diagnostic criteria for probable sarcopenia.^40^, ^46^

Our study supports the cut‐off score of 7/8 for cognitive impairment. The recommended cut‐off for RCS for cognitive impairment is 7, which can be applied to our local multi‐ethnic older population as 58.1% either have no formal education or received ≤6 years of education.^17^, ^47^ Apart from the cross‐sectional nature of our study, limitations include the use of the MoCA as a reference screening tool for cognitive impairment rather than a full neuropsychological assessment with application of appropriate diagnostic criteria. However, the performance of the MoCA in distinguishing cognitive impairment from normal cognition has been widely studied and validated.^38^ Our study also relied heavily on self‐report, which may lead to recall bias, especially in those with underlying cognitive impairment. While we managed to establish validity and reliability of RCS against MoCA, we have no information in specific groups including those with vascular cognitive impairment or Parkinson's disease. Our sample size was small to study the effect of education on RCS cut‐off in the old and old‐old subgroup. Finally, we did not get additional collaborative histories from caregivers to support diagnoses of cognitive impairment.

## CONCLUSION

The three‐item RCS is quick and easy to administer. Although RCS met the criterion for good validity against MoCA in predicting cognitive impairment, its utility as a first‐line screening tool needs to be further validated in a large‐scale population study and in different subgroups such as Parkinson's disease and vascular cognitive impairment.
